# Supplementary material for: Mechanical Rupture-Based Antibacterial and Cell-Compatible ZnO/SiO2 Nanowire Structures Formed by Bottom-Up Approaches
Source: Micromachines (Basel). 2020 Jun 24;11(6):610. doi: 10.3390/mi11060610 (PMC7345559; doi:10.3390/mi11060610)
Supplement: Supplementary file 1 [file micromachines-11-00610-s001.pdf]

Supplementary Materials: Mechanical Rupture-Based Antibacterial and Cell-Compatible ZnO/SiO2 Nanowire Structures Formed by Bottom-Up Approaches

Taisuke Shimada, Takao Yasui, Akihiro Yonese, Takeshi Yanagida, Noritada Kaji, Masaki Kanai, Kazuki Nagashima, Tomoji Kawai and Yoshinobu Baba

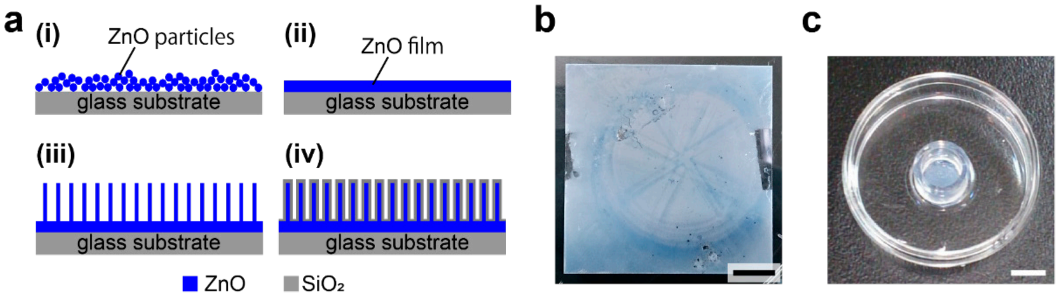

Figure S1. (**a**) Scheme of fabrication process: (i) spin-coating of seeding solution and deposition of ZnO particles; (ii) formation of ZnO film by heating treatment; (iii) nanowire growth via hydrothermal synthesis; and (iv) SiO_2 deposition on the ZnO nanowire surface by atomic layer deposition. Photographs of ZnO/SiO_2 nanowire substrate for: (**b**) the antibacterial test and (**c**) the cell viability test (scale bars, 1 cm).

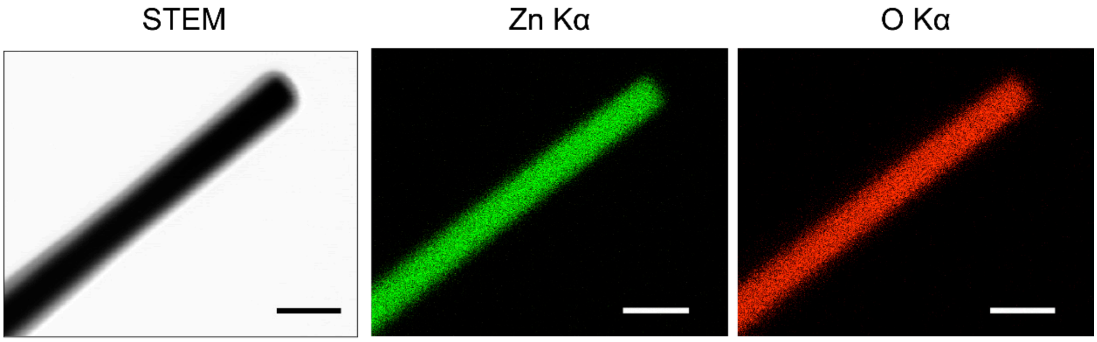

Figure S2. Elemental mapping images of a single ZnO nanowire. Images of STEM, Zn *Kα* and O *Kα* are shown here (scale bars, 100 nm).

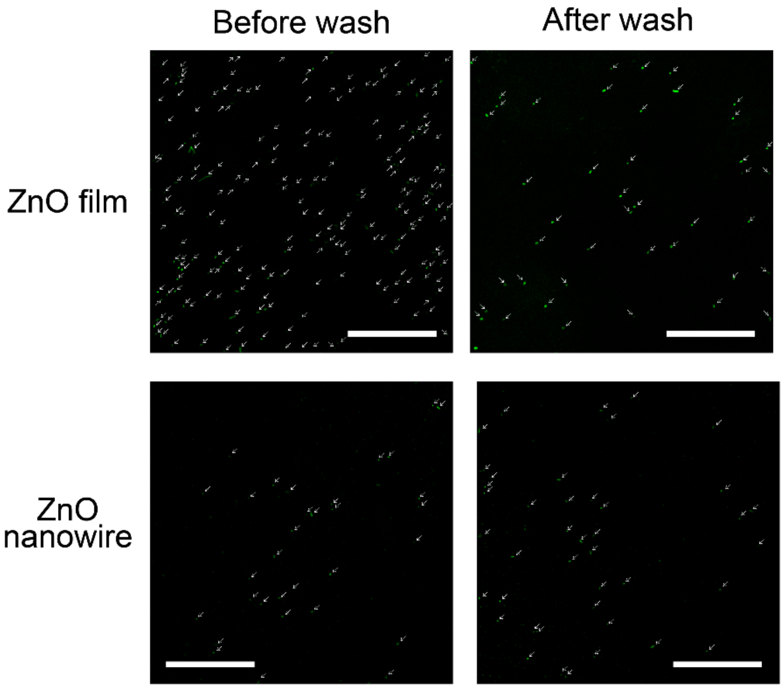

Figure S3. Fluorescence images of ZnO film and ZnO nanowire substrate for the antibacterial test before and after washing out (scale bars, 100 μm).
